# FORECASTING THE FUTURE: OLDER ADULTS’ ANTICIPATED STRESSORS AND REWARDS IN THE CONTEXT OF COVID-19

**DOI:** 10.1093/geroni/igad104.0241

**Published:** 2023-12-21

**Authors:** Christina Marini, Erin Basinger, Meagan Stewart, Katherine Fiori, Amy Rauer

**Affiliations:** Adelphi University, Garden City, New York, United States; University of North Carolina at Charlotte, Charlotte, North Carolina, United States; University of Tennessee, Knoxville, Tennessee, United States; Adelphi University, Garden City, New York, United States; University of Tennessee, Knoxville, Tennessee, United States

## Abstract

Stress research typically focuses on past/current events as opposed to future ones. We identified future anticipated stressful and rewarding events at 2 timepoints utilizing qualitative data from older adults (N = 103, Mage = 68.11, 68.9% female, 93.2% white) who participated in a study of social reintegration after vaccines were available in the US. COVID-19 cases were typically below 1 million during T1 (Fall 2021), but exceeded 5 million during T2 (Winter 2022), coinciding with the Omicron variant. This afforded the opportunity to examine anticipated stressors and rewards at timepoints with different levels of COVID-19 threat. We used content analysis to identify anticipated stressors and rewards, which fit into 13 categories (Cohen’s κ = .86-.96) and examined rates of ambivalence (i.e., same event was a stressor and reward within timepoints) and chronicity (i.e., same stressor or reward was named across timepoints). At T1, the modal stressor was own transition/change (13.6%; e.g., relocating). At T2, it was the pandemic (20.4%; e.g., family member recovering from COVID). Family/friend relationships (e.g., celebrating grandkids’ birthdays) was the modal reward at T1 (32%) and T2 (24.3%). A quarter of participants demonstrated ambivalence (T1 = 23.3%; T2 = 28.2%). Stressor and reward chronicity (22.3% and 32%, respectively) were low. Findings highlight variability in the events older adults were concerned and excited about within and across timepoints. Salience of the pandemic as an anticipated stressor ebbed and flowed alongside emerging variants, despite vaccine availability.

